# Multiomics analysis of *Hemsleya ellipsoidea* reveals genome evolution and specialized cucurbitacin IIa biosynthesis in a medicinal Cucurbitaceae species

**DOI:** 10.1093/hr/uhaf363

**Published:** 2026-01-13

**Authors:** Fei-Fan Zhao, Gui-Chao Xie, Li-Ming Huang, Kai-Heng Chen, Wen-Li Zhang, Yu-Mei Feng, Zhi-Kuan Wang, Peng Zhang, Zhi-Chao Qiao, Chun-Hua Fu, Long-Jiang Yu

**Affiliations:** Institute of Resource Biology and Biotechnology, Department of Biotechnology, College of Life Science and Technology, Huazhong University of Science and Technology, Wuhan 430074, China; Key Laboratory of Biophysics in the Education Department; Hubei Engineering Research Centre for Dual-use Resource Development of Food and Medicine, Wuhan 430074, China; Institute of Resource Biology and Biotechnology, Department of Biotechnology, College of Life Science and Technology, Huazhong University of Science and Technology, Wuhan 430074, China; Key Laboratory of Biophysics in the Education Department; Hubei Engineering Research Centre for Dual-use Resource Development of Food and Medicine, Wuhan 430074, China; Institute of Resource Biology and Biotechnology, Department of Biotechnology, College of Life Science and Technology, Huazhong University of Science and Technology, Wuhan 430074, China; Key Laboratory of Biophysics in the Education Department; Hubei Engineering Research Centre for Dual-use Resource Development of Food and Medicine, Wuhan 430074, China; Yunnan Shanyuan Biotechnology Co., Ltd., Lincang, Yunnan 677000, China; Institute of Resource Biology and Biotechnology, Department of Biotechnology, College of Life Science and Technology, Huazhong University of Science and Technology, Wuhan 430074, China; Key Laboratory of Biophysics in the Education Department; Hubei Engineering Research Centre for Dual-use Resource Development of Food and Medicine, Wuhan 430074, China; Institute of Resource Biology and Biotechnology, Department of Biotechnology, College of Life Science and Technology, Huazhong University of Science and Technology, Wuhan 430074, China; Key Laboratory of Biophysics in the Education Department; Hubei Engineering Research Centre for Dual-use Resource Development of Food and Medicine, Wuhan 430074, China; Institute of Resource Biology and Biotechnology, Department of Biotechnology, College of Life Science and Technology, Huazhong University of Science and Technology, Wuhan 430074, China; Key Laboratory of Biophysics in the Education Department; Hubei Engineering Research Centre for Dual-use Resource Development of Food and Medicine, Wuhan 430074, China; Institute of Resource Biology and Biotechnology, Department of Biotechnology, College of Life Science and Technology, Huazhong University of Science and Technology, Wuhan 430074, China; Key Laboratory of Biophysics in the Education Department; Hubei Engineering Research Centre for Dual-use Resource Development of Food and Medicine, Wuhan 430074, China; Institute of Resource Biology and Biotechnology, Department of Biotechnology, College of Life Science and Technology, Huazhong University of Science and Technology, Wuhan 430074, China; Key Laboratory of Biophysics in the Education Department; Hubei Engineering Research Centre for Dual-use Resource Development of Food and Medicine, Wuhan 430074, China; Institute of Resource Biology and Biotechnology, Department of Biotechnology, College of Life Science and Technology, Huazhong University of Science and Technology, Wuhan 430074, China; Key Laboratory of Biophysics in the Education Department; Hubei Engineering Research Centre for Dual-use Resource Development of Food and Medicine, Wuhan 430074, China; Institute of Resource Biology and Biotechnology, Department of Biotechnology, College of Life Science and Technology, Huazhong University of Science and Technology, Wuhan 430074, China; Key Laboratory of Biophysics in the Education Department; Hubei Engineering Research Centre for Dual-use Resource Development of Food and Medicine, Wuhan 430074, China

## Abstract

*Hemsleya ellipsoidea* (Xuedan) is a phylogenetically distinct medicinal species within the Cucurbitaceae family, notable for its ability to accumulate cucurbitacin IIa—a bioactive triterpenoid with potent anti-inflammatory and antibacterial activities. Here, we present a chromosome-scale reference genome for *H. ellipsoidea*, assembled using Oxford Nanopore, Illumina, and Hi-C sequencing technologies. The 535.68 Mb genome, with a contig N50 of 15.36 Mb, encodes 25 230 protein-coding genes across 14 pseudo-chromosomes, of which 63.85% comprise repetitive elements. Comparative genomic and phylogenomic analyses reveal that *H. ellipsoidea* diverged early (~84.7 MYA) from other cucurbits, maintaining several ancestral chromosomal segments but exhibiting lineage-specific rearrangements, reflecting an independent evolutionary trajectory without recent whole-genome duplication. Two conserved but functionally specialized biosynthetic gene clusters related to cucurbitacins formation were identified, suggesting coordinated regulation of triterpenoid metabolism. Integration of genomic and transcriptomic data enabled the reconstruction of the cucurbitacin IIa biosynthetic pathway and the identification of key structural enzymes and transcription factors. Distinct tissue-specific expression patterns further indicate root-localized synthesis and accumulation of cucurbitacin IIa. Collectively, this work provides the first high-quality genome of a medicinal Cucurbitaceae species and offers new insights into the chromosomal evolution, metabolic specialization, and adaptive diversification of *H. ellipsoidea*. The genomic resource also lays a foundation for functional genomics, metabolic engineering, and molecular breeding toward high-value triterpenoid production.

## Introduction

*Hemsleya ellipsoidea* (Xuedan, 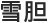) is a perennial, non-woody climbing plant in the Cucurbitaceae family (2*n* = 28), primarily distributed in southern China. Over 30 species of *Hemsleya* have been reported in China and Vietnam. The tuberous roots of *Hemsleya* plants are widely used in traditional Chinese medicine. These roots are valued for their bitterness and ‘cold’ properties, which are traditionally believed to detoxify the body and dispel internal heat. In addition to their reported activities against HIV and tumors, species within this genus have been used to treat inflammatory conditions, such as bacillary ulcers and jaundice [1–3]. Consequently, several species, including *H. chinensis* [4], *H. amabilis* [5], *H. graciliflora*, and *H. macrosperma* [6] possess significant anti-inflammatory properties and are extensively developed for clinical practice. However, the surging demand for these medicinal materials, coupled with the depletion of wild populations, has precipitated a severe resource shortage. Among the utilized species, *H. ellipsoidea* is particularly distinguished by its exceptionally high synthesis and accumulation of bioactive compounds, making it a strategic target for relieving resource pressure.

The major bioactive constituents of *H. ellipsoidea* are triterpenoid saponins, specifically cucurbitacins, which accumulate predominantly in the tuberous roots [7]. These highly oxygenated tetracyclic triterpenes are largely responsible for the pharmacological activities [8–11] and characteristic bitterness of the genus [12]. Compared to other *Hemsleya* species, *H. ellipsoidea* exhibits a superior capacity for cucurbitacin accumulation. Selective breeding efforts have led to elite cultivars with cucurbitacin IIa (CuIIa) content exceeding 2.5%. However, cultivation remains challenging due to slow growth (3–5 years to harvest), high costs, and strict ecological requirements. To ensure stable and sustainable production of cucurbitacins, a deeper understanding of their biosynthetic pathways is urgently needed. Therefore, elucidating the biosynthetic pathway and regulatory mechanisms of cucurbitacins has become an urgent and essential objective.

Cucurbitacins are a subclass of highly oxidized tetracyclic triterpenoids, mainly synthesized via the mevalonate (MVA) and methylerythritol phosphate (MEP) pathways [13]. Cucurbitacins exhibit remarkable structural diversity and are currently classified into 12 major types, designated as Cucurbitacins A through T [14]. *H. ellipsoidea* specifically produces the pharmaceutically active ingredients CuIIa and cucurbitacin IIb, with CuIIa constituting a significant majority [15]. Common triterpenoids biosynthesis originates from key precursors of isopentenyl diphosphate (IPP) and dimethylallyl diphosphate (DMAPP). The MVA pathway and MEP pathway generate these two precursors. Then squalene was generated by the consecutive catalysis of IPP and DMAPP together. Squalene is subsequently oxidized to form 2,3-oxidosqualene by squalene epoxidase (*SE*). The pathway then reaches the branch point of steroids and triterpenoids following catalysis by oxidosqualene cyclases (OSCs) [16]. The product varies due to different kinds of *OSCs* and could be modified through cytochrome P450 monooxygenases (CYP450s) [17], UDP-glycosyltransferases (UGTs) [18], acyltransferases (ACTs) [12], and methyltransferases (MTs).

In model cucurbits (e.g., cucumber, melon, bitter melon), the biosynthesis of cucurbitacins has been well characterized, with several key CYP450s and OSCs identified. In *Citrullus lanatu*s (watermelon) and *Cucumis melo* (melon), cucurbitadienol (Cuol) serves as the triterpene backbone, and studies have shown that *CYP81Q59* (e.g., *Cm180* and *Cl180*) catalyzes hydroxylation at the C2 position, while *CYP87D20* (e.g., *Cm890* and *Cl890*) is responsible for oxidation at C11 and hydroxylation at C20 [19]. Homologs of these genes have also been identified in the transcriptome of *H. chinensis* and experimentally validated to exhibit similar catalytic activities [20]. In *Cucumis sativus* (cucumber), *CsCYP88L2* and *CYP81Q58* mediate hydroxylation at C19β and C25, respectively [12]. In *Momordica charantia* (bitter melon), *CYP88L8* catalyzes hydroxylation at C7, *McCYP88L7* is involved in the formation of a C5 – C19 ether bridge and hydroxylates C19, while *McCYP81AQ19* catalyzes hydroxylation at C23 [21]. In the noncucurbitaceous species *Iberis amara*, *CYP708A16* and *CYP708A15* are responsible for C16β and C22 hydroxylation, respectively, using Cuol-based substrates [22]. These findings highlight the diversity of CYP450-mediated modifications and offer a reference framework for pathway reconstruction in non-model species. However, these studies have primarily focused on fruit-bearing cucurbits, which accumulate cucurbitacins in aerial organs. In contrast, *H. ellipsoidea* is a root-specialized, medicinal cucurbit, offering a unique perspective into organ-specific triterpenoid biosynthesis. Despite its pharmacological importance, the lack of a reference genome has hindered the molecular understanding of *H. ellipsoidea*. Without a high-quality genome assembly, the systematic identification of biosynthetic gene clusters (BGCs), lineage-specific gene family expansions, and regulatory elements involved in CuIIa biosynthesis has been impossible. This gap severely limits molecular breeding, metabolic engineering, and conservation of this valuable medicinal resource.

With the progress and cost reduction of sequencing technologies, a large number of genomes from cucurbitaceous plant species have been sequenced in recent years [23], including the tribes of Cucurbiteae [24], Sicyoeae, Siraitieae [25], Benincaseae [26], and Momordiceae [27], facilitating gene identification, genomic evolution, and genetic variation studies in the cucurbit family. Whole-genome sequencing has become a crucial tool in the field of natural product biosynthesis [28]. And next-generation sequencing (NGS) technologies such as Oxford Nanopore Technologies (ONT) have enabled accurate genome assembly [29], offering valuable insights into the genetic basis and regulatory mechanisms of these compounds. Coupling high-quality genome data with transcriptomic and phylogenomic analyses allows for the identification of gene clusters, metabolic modules, and regulatory networks underpinning medicinal compound biosynthesis.

In this study, we generated the first chromosome-level genome assembly of *H. ellipsoidea*, representing the first such resource for any medicinal Cucurbitaceae species. Our aim was to characterize the genomic basis of CuIIa biosynthesis and investigate how genomic rearrangements, gene family evolution, and co-expression networks contribute to specialized triterpenoid metabolism. This work fills a major gap in cucurbit genomic resources and lays the foundation for molecular breeding and metabolic engineering of *Hemsleya* and related medicinal plants.

## Results

### Genome assembly

**Figure 1:**
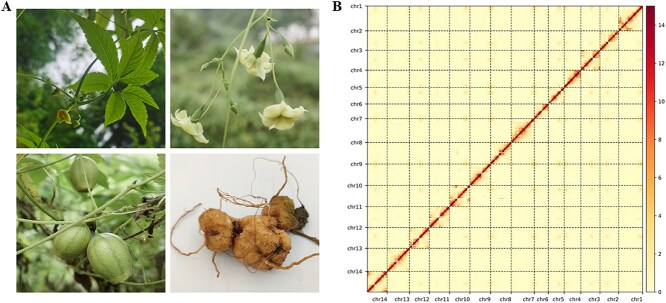
Overview of the *H. ellipsoidea* and its genome. (A): Different organs of *H. ellipsoidea*. (B): Interactions of the 14 pseudochromosomes obtained by Hi-C in the *H. ellipsoidea* genome.

To gain genomic insights into *H. ellipsoidea*, a medicinally important species endemic to Southwest China (Fig. 1A), three different sequencing methods were employed to obtain its genome data. Firstly, a total of 56.13Gb of raw data with 150 bp insert libraries, was generated by Illumina NovaSeq 6000 sequencing, and 56.07 Gb of clean data was obtained after data filtering (Table S1). The depth frequency distribution analysis was conducted using k-mer analysis (*k* = 19). Based on this analysis, the genome size of *H. ellipsoidea* was estimated at 537.31 Mb, characterized by high heterozygosity (1.62%) and repetition (59.06%), which suggests that the genome assembly is particularly complex. (Fig. S1 and Table S2).

Then 60.45Gb of Oxford Nanopore sequencing data was generated to obtain the assembly (Table S3). Based on the sequencing data from the two platforms, we then used 60.01Gb of raw data from the Hi-C library (coverage of 100X) to construct physical maps. The assembled scaffolds were clustered into 14 pseudochromosomes, indicating that the genome contains 14 chromosomes (Table S4). At last, 64280570935 bp sequences were mapped to the 14 chromosomes accounting for 91.11% of the raw data. The N50 value was 14.7 M, with the length of the 14 pseudochromosomes ranging from 26.54 M to 44.09 M (Table 1). In addition, the BUSCO estimation demonstrated that the assembled genome achieved a completeness level of 95.60%. Consistently, when clean short reads were mapped back to the assembly, it was revealed that the corresponding completeness reached 99.71% (Tables S5 and S6).

**Figure 2:**
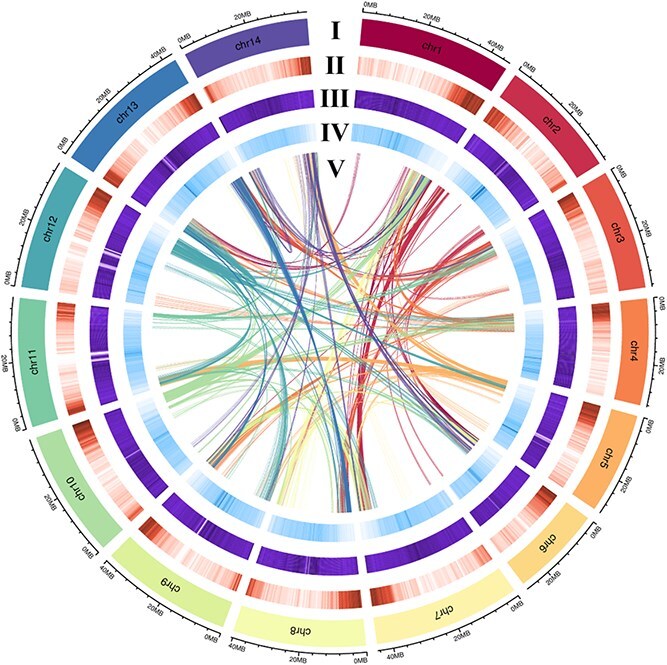
Distribution of *H. ellipsoidea* genomic features. (I) Circular representation of the pseudomolecule. (II–IV) gene density (500 kb window), percentage of repeats (500 kb window), and GC content (500 kb window). (V) Each linking line in the center of the circle connects a pair of homologous genes.

The Hi-C heatmap of the assembled genome chromosomes of *H. ellipsoidea* showed that high interaction intensity was observed within each chromosome, with no abnormal signals (Fig. 1B), confirming the accuracy and high confidence of the Hi-C assisted assembly for the genome. A comprehensive circos plot illustrates the genomic landscape of *H. ellipsoidea*, including gene density, GC content, and repeat distribution across all 14 pseudo-chromosomes (Fig. 2).

### Genome annotation

The genome of *H. ellipsoidea* was annotated using an integrative strategy that combined de novo, homology-based, and transcriptome-assisted predictions. A total of 25 230 genes, 127 069 exons, and 107 839 introns were identified (Table S7). Among them, 99.98% of coding regions (≥50% coverage) overlapped between the *de novo* and homology predictions, with 95.7% exhibiting high completeness (Table S8). The average gene size was 4985 bp, with 5.34 exons per gene. The total number of protein-coding genes (23 803) is comparable to that of other Cucurbitaceae species such as the bottle gourd (22472) [30], cucumber (23 248) [31], and watermelon (23 440) [32]. In total, 95.43% of genes were functionally annotated in public databases, confirming the completeness and reliability of the assembly. While the overall gene count is similar to that of other cucurbits, *H. ellipsoidea* exhibited shorter exon and intron lengths, suggesting a more compact gene architecture possibly associated with metabolic specialization for CuIIa biosynthesis (Fig. S2).

**Table 1:** Genome assembly statistics of *H. ellipsoidea*

**Items**	**Values**
Total_length(bp)	535 676 299
Total_length_withoutN(bp)	535 676 299
Total_number	73
GC_content(%)	33.61
N50(bp)	15 416 820
N90(bp)	4 826 948
Average(bp)	7338031.49
Median(bp)	4826948.00
Min(bp)	147 826

A total of 509 tRNAs, 120 miRNAs, 266 rRNAs, and 623 snRNAs were identified (Table S10). Repetitive elements accounted for 63.85% of the genome, lower than the 75.5% observed in the wax gourd (*Benincasa hispida*) genome. Among these repeats, 33.6% were annotated as long terminal repeat (LTR) retrotransposons, predominantly Copia-type (17.58%) and Gypsy-type (14.49%) LTRs [32, 33]. Compared with other Cucurbitaceae species, the relatively reduced repeat content and compact genic regions indicate that *H. ellipsoidea* has undergone fewer recent transposon bursts, maintaining a stable and specialized genome structure. Such genomic compactness may have facilitated the co-regulation of secondary metabolic genes, particularly those involved in triterpenoid (CuIIa) biosynthesis, highlighting the evolutionary adaptation of this medicinal lineage within the Cucurbitaceae family.

### Comparative genomic analysis and phylogeny of *H. ellipsoidea*

Comparative genomic analysis was conducted between *H. ellipsoidea* and ten sequenced species, including seven Cucurbitaceae family members (*C. melo, Cucurbita pepo, C. sativus, C. moschata, C. maxima, B hispida,* and *M. charantia*), as well as *Vitis vinifera, Oryza sativa,* and *Arabidopsis thaliana*. Across all species, 43 794 orthologous gene families encompassing 174 714 genes were identified, among which 6995 were conserved gene families and 1401 were single-copy gene families. Notably, the *H. ellipsoidea* genome contained 2243 unique gene families, including 4476 lineage-specific paralogs, suggesting substantial species-specific gene diversification.

**Figure 3:**
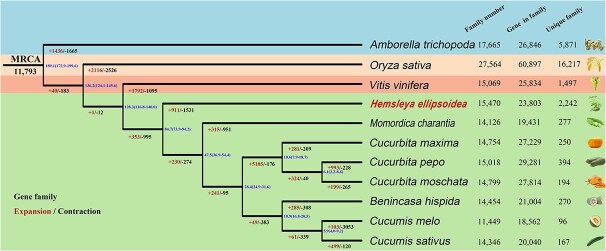
Phylogenetic relationships and comparative genomic analyses.

A phylogenetic tree constructed using 3768 single-copy orthologs (Figs 3 and Fig. S3) showed that *H. ellipsoidea* diverged from the other Cucurbitaceae species approximately 84.7 million years ago (MYA) (Fig. S4), indicating an early and independent evolutionary trajectory within the family. Gene family expansion and contraction analyses (Fig. S5) revealed that 911 families were expanded and 1531 contracted in *H. ellipsoidea*. GO and KEGG enrichment analyses of the expanded families demonstrated significant overrepresentation of genes associated with ‘oxidoreductase activity’, ‘membrane’, and ‘extracellular region’ (Fig. S6), as well as ‘ribosome’ and several metabolic processes (Fig. S8). These categories are functionally relevant to secondary metabolite biosynthesis and cellular detoxification.

Further comparison among *H. ellipsoidea*, *C. pepo*, *C. sativus*, and *B. hispida* revealed 2567 gene families unique to *H. ellipsoidea* (Fig. S10). GO enrichment of these genes indicated significant enrichment in hydrolase activity and ADP-binding functions, while KEGG analysis revealed enrichment in ‘amino sugar and nucleotide sugar metabolism’ and ‘terpenoid backbone biosynthesis’ (Figs S11 and S12). The latter pathway showed moderate but statistically significant enrichment, supporting the notion that *H. ellipsoidea* has evolved distinct genomic features linked to triterpenoid biosynthesis, particularly the formation of CuIIa. Collectively, these results highlight the unique evolutionary status of *H. ellipsoidea* within the Cucurbitaceae family and its specialization toward complex triterpenoid metabolism. The results provide a high-resolution evolutionary framework for Cucurbitaceae comparative genomics. The implementation of universal phylogenomic probe sets (e.g., Angiosperms353) [34] in recent studies further illustrates the growing importance of standardized genomic integration in resolving complex lineage relationships and validating orthogroup-based evolutionary reconstructions.

A phylogenetic tree was constructed using 12 plant species to investigate gene family evolution. The blue numbers adjacent to each node indicate the estimated divergence times (MYA, million years ago). For each species, three parameters are shown: the number of gene families, the number of genes assigned to these families, and the total number of annotated genes.

### Whole-genome duplication and synteny analysis

Whole-genome duplication (WGD) is a key evolutionary process in angiosperms, expanding gene repertoires and driving functional diversification through sub- and neofunctionalization [35]. Polyploidy enhances genomic plasticity and adaptive potential, often facilitating lineage-specific innovations.

**Figure 4:**
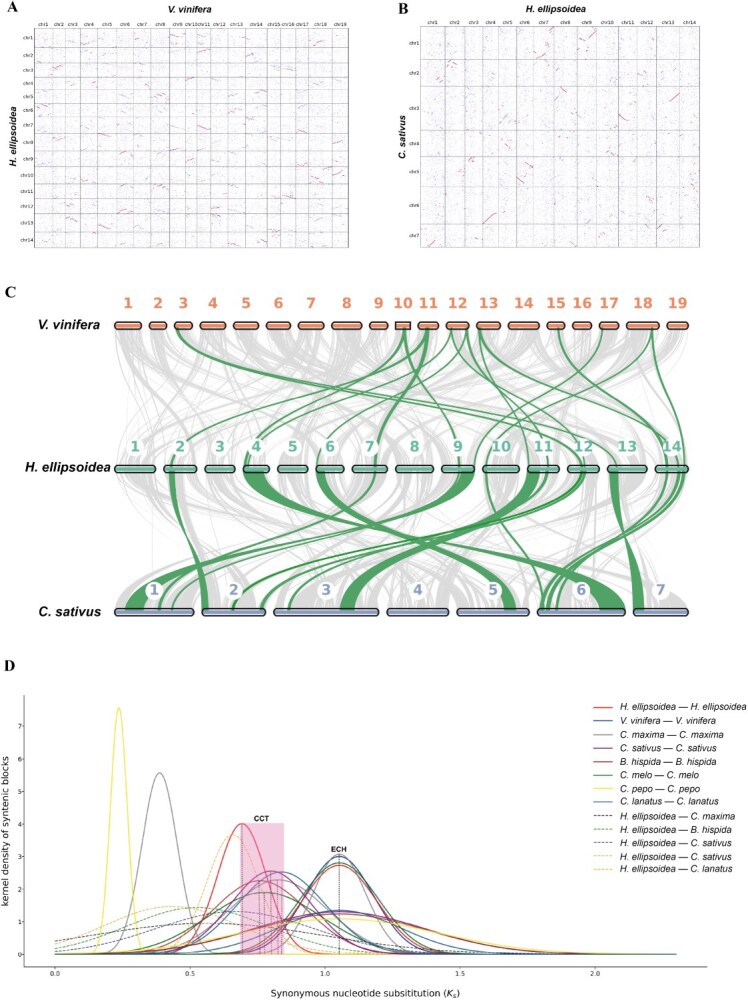
Comparative genomics analyses. (A): dotplot analysis between *H. ellipsoidea* and *V. vinifera*. The red circles indicate that the chromosomes of V. vinifera exhibit a 1:2 syntenic relationship with *H. ellipsoidea*. (B): Dotplot analysis between *H. ellipsoidea* and *C. sativus*. There is only 1:1 syntenic relationship between the two species. (C): Macrosynteny of *V. vinifera*, *H. ellipsoidea,* and *C. sativus* karyotypes. (D): distribution of synonymous substitution levels (Ks) of syntenic orthologous and paralogous genes.

To investigate the evolutionary trajectory of *H. ellipsoidea*, we conducted comparative genomic and syntenic analyses across representative Cucurbitaceae species, including *C. melo*, *C. pepo*, *C. sativus*, *C. moschata*, *B. hispida*, *C. maxima*, *M. charantia*, and *C. lanatus*. The synteny comparison between *H. ellipsoidea* and grape (*V. vinifera*) revealed a clear 2:1 correspondence (Fig. 4A), indicating a lineage-specific duplication event in *H. ellipsoidea*. Similar 2:1 patterns were detected in other cucurbits (Figs S13–S17), while *Cucurbita* species displayed a 4:1 relationship (Figs S18–S20), suggesting an additional WGD within that genus.

Synteny between *H. ellipsoidea* and other cucurbits showed two major correspondence patterns (Fig. 4B and C**;**  Figs S21–S27). Previous studies have documented two ancient duplication events in eudicots—the Eudicot common hexaploidy (ECH) [36] and the Cucurbitaceae common tetraploidization (CCT) [37]. Our results confirm that *H. ellipsoidea*, like other cucurbits, has undergone both the ECH and the CCT. However, *H. ellipsoidea* exhibits extensive chromosomal fissions and fusions following the CCT, resulting in a markedly restructured karyotype compared with conserved species such as *B. hispida*. Our results confirm that the Cucurbitaceae family has undergone both of these events. The older WGD event corresponds to the ECH, whereas the more recent event aligns with the CCT. Additionally, our analysis indicates that the genus *Cucurbita* experienced an additional WGD event, distinguishing it from other Cucurbitaceae species.

The analysis of synonymous substitution rates (Ks) for collinear gene pairs further supports this evolutionary pattern (Fig. 4D). Using *V. vinifera* as an outgroup, we traced the ECH event and refined duplication histories across the Cucurbitaceae. Divergence time estimates indicate that the Cucurbitaceae lineage diverged synchronously after ECH, but *H. ellipsoidea* separated earlier from other cucurbits and has not experienced any recent WGD events. This independent chromosomal evolution may have contributed to its unique biosynthetic specialization, including the formation of CuIIa—a hallmark metabolite absent from other cucurbit species.

### Karyotype evolution of *H. ellipsoidea* within the Cucurbitaceae Family

**Figure 5:**
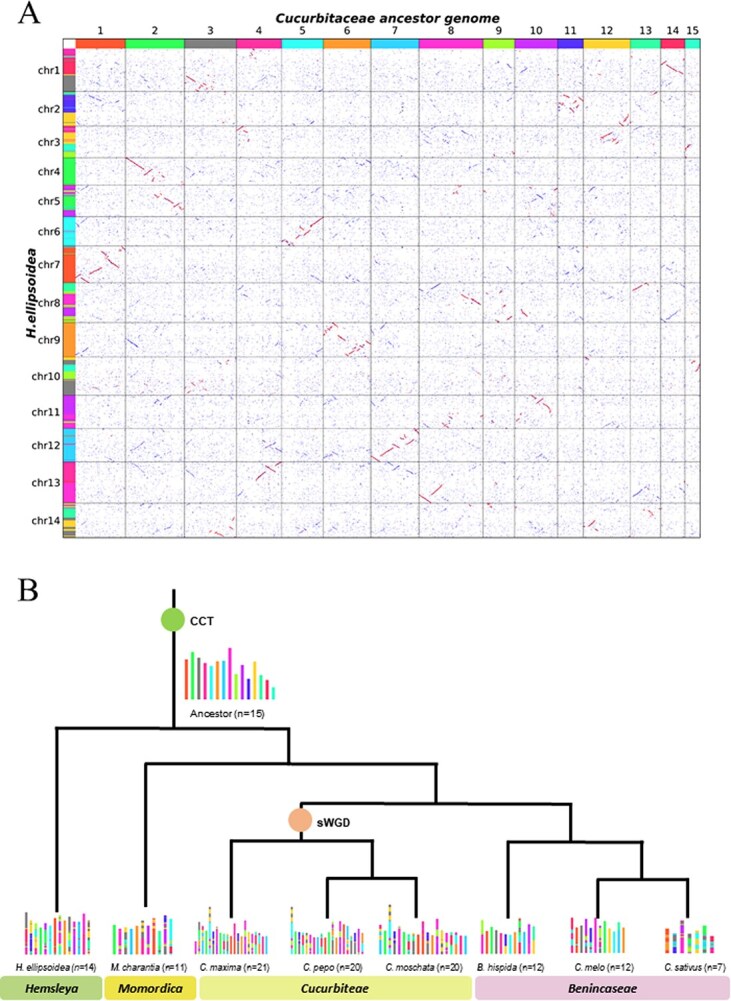
Evolutionary scenario of the *H. ellipsoidea* genome from the ancestral Cucurbitaceae karyotype. (A) Syntenic dot plot between the genome of *H. ellipsoidea* and the reconstructed ancestral Cucurbitaceae genome. Each colored dot represents a shared collinear gene pair, and identical colors indicate conserved chromosomal segments. (B) Evolutionary trajectory of Cucurbitaceae karyotype evolution. The CCT event denotes the Cucurbitaceae-common tetraploidization, and sWGD indicates a lineage-specific WGD event in species of the tribe Cucurbiteae.

To elucidate the chromosomal evolution of *H. ellipsoidea*, we reconstructed the ancestral karyotype of the Cucurbitaceae based on the method proposed by Xie et al. [26], using the latest high-quality genome of *B. hispida* (version pf3, released in 2023) as a reference [38]. Compared with the earlier assembly (B227, released in 2019), the pf3 genome provides a more complete and accurate foundation for ancestral inference. The ancestral karyotype, corresponding to the post-CCT state, was inferred using high-resolution collinearity analysis with WGDI. A detailed synteny comparison was then performed between the ancestral chromosomes and those of *H. ellipsoidea* (Fig. 5).

Quantitative analysis of syntenic relationships identified a total of 118 synteny blocks linking the *H. ellipsoidea* genome to the ancestral Cucurbitaceae karyotype (Table S11). As visualized in the synteny map (Fig. 5A), we categorized the *H. ellipsoidea* chromosomes into three distinct evolutionary patterns based on their rearrangement frequency. First, chromosomes 4, 7, 9, and 12 are highly conserved, largely preserving the ancestral structure. Notably, Chr 9 represents the most stable lineage, consisting of a single synteny block (100% collinearity) derived entirely from Ancestral Chr 6. Second, in sharp contrast, chromosomes 1, 2, 3, 5, 8, 11, and 14 act as complex fusion chromosomes, having undergone extensive fragmentation and fusion. Chr 2 exhibits the highest degree of shattering, comprising 16 distinct synteny blocks derived primarily from Ancestral Chr 11 but fused with segments from six other ancestral sources. Similarly, Chr 5, 8, 11, and 14 each contain 10–12 blocks, reflecting high plasticity. Finally, the remaining chromosomes (e.g., Chr 6, 10, 13) show intermediate rearrangement levels. Chr 10, specifically, is highly fragmented (8 blocks), indicating potential gene loss or localized inversions.

To assess broader conservation patterns, we mapped the 15 reconstructed ancestral chromosomes across representative species from *Momordica*, *Cucurbita*, and *Benincasa*, and visualized their retention on the phylogenetic framework (Fig. 5B). *H. ellipsoidea* retained a higher proportion of ancestral chromosomal segments than most other cucurbits, second only to *B. hispida* and *C. melo*. This confirms that *B. hispida* represents the most conserved karyotype within the family, whereas *H. ellipsoidea* experienced pronounced chromosomal reshuffling after the CCT event. Such structural reorganization, characterized by extensive translocations and segmental losses, reflects lineage-specific genomic remodeling in *H. ellipsoidea*. These chromosomal innovations may have provided the structural basis for metabolic diversification, particularly the evolution of the CuIIa biosynthetic pathway unique to this medicinal species.

### Identification of genes and gene clusters involved in triterpenoid biosynthesis

**Figure 6:**
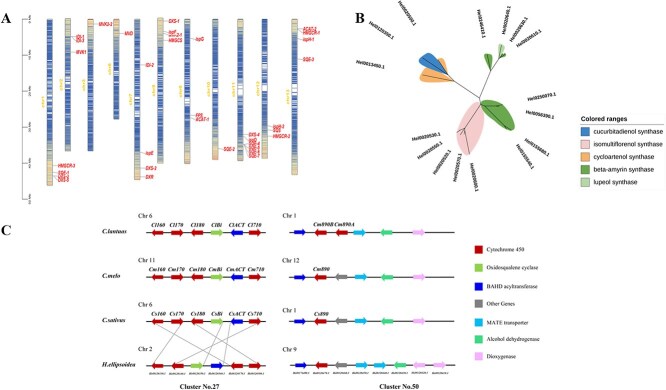
Triterpenoid biosynthetic genes and gene clusters in *H. ellipsoidea*. (A) Chromosomal distribution of key genes involved in the MVA and MEP pathways and triterpenoid biosynthesis. Genes are broadly distributed across 13 chromosomes, with no relevant loci identified on chromosome 7. (B) Phylogenetic analysis of the OSC gene family in *H. ellipsoidea*, showing classification into distinct clades corresponding to predicted triterpenoid skeleton types. (C) Synteny comparison of Cluster No. 27 and No. 50 with homologous triterpenoid-related gene clusters in closely related Cucurbitaceae species, highlighting conserved collinear arrangements.

To elucidate the genetic basis underlying triterpenoid biosynthesis in *H. ellipsoidea*, we conducted a comprehensive genome-wide identification and functional annotation of genes involved in the MVA and MEP pathways using multiple complementary approaches. A total of 25 genes were identified across these two pathways (Table S12). Among them, *HMGCS*, *MVK1*, and *MVD* were found to be single-copy genes in the MVA pathway, while *ispD*, *ispE*, *ispF*, and *ispG* were identified as single-copy genes in the MEP pathway. Furthermore, chromosomal mapping of these genes revealed that they are distributed across most chromosomes, with the exception of chromosomes 4, 5, and 14 (Fig. 6A). This widespread genomic distribution implies that triterpenoid biosynthetic genes are not physically clustered but rather dispersed throughout the genome, potentially reflecting the independent regulation of distinct enzymatic steps.

We further identified key genes involved in triterpenoid skeleton biosynthesis by extending the same analytical framework, including one farnesyl diphosphate synthase (*FDPS*), one squalene synthase (*SQS*), seven squalene epoxidase (*SQLE*) genes, and sixteen candidate *OSC* genes. Among them, *FDPS* and *SQS* were found to be single-copy genes, whereas *SQLE* was present in multiple copies, indicating potential functional diversification. Further domain annotation and sequence homology analysis of the *OSC* candidates revealed their putative roles in catalyzing the formation of five distinct triterpenoid skeletons. Specifically, we identified a single-copy gene encoding Cuol synthase (*Hel0120350.1*) and a single-copy gene encoding lupeol synthase (*Hel0020640.1*), suggesting their specialized roles in cucurbitacin and lupeol biosynthesis, respectively. In addition, seven genes were annotated as β-amyrin synthases, five as isomultiflorenol synthases, and two as cycloartenol synthases. These functional predictions were further supported by a phylogenetic analysis of the OSC proteins, which clustered into five distinct clades corresponding to the proposed catalytic products (Fig. 6B).

In recent years, metabolic gene clusters (MGCs) have been widely recognized as key genomic units that facilitate the efficient biosynthesis and coordinated regulation of lineage-specific specialized metabolites in plants [39, 40]. Building upon the identification of core biosynthetic enzymes, we further performed a systematic analysis of secondary metabolism-related gene clusters in *H. ellipsoidea*. Using the high-quality genome assembled in this study and applying the antiSMASH [41], we identified a total of 50 candidate MGCs associated with the biosynthesis of various classes of specialized metabolites, including terpenoids, flavonoids, and alkaloids (Table S13). These gene clusters were distributed across all chromosomes except chromosome 7, suggesting a degree of chromosomal functional compartmentalization, with chromosome 7 likely being enriched for primary metabolic or structural functions. Among the predicted clusters, nine were identified as terpenoid-related MGCs (Tables S14–S23), harboring key genes involved in the MVA and MEP pathways, members of the CYP450 superfamily, all identified *OSC* genes, and several BAHD acyltransferases. Genes from the MVA pathway were found in seven gene clusters, underscoring their pervasive involvement in the triterpenoid biosynthetic network. Notably, cluster No. 27 contained a full complement of key genes required for cucurbitacin IIA biosynthesis, including an experimentally validated Cuol synthase (*HeOSC*), a BAHD-acyltransferase (*HeACT*), and four CYP450 genes (Fig. 6C). Among the four CYP450 genes, *Hel0120330.1* shares high sequence similarity with *Cs170*, *Cm170*, and *Cl170*, and is annotated as *CYP89A140*. *Hel0120340.1* belongs to the CYP87D19 subfamily and exhibits strong homology to *Cs710*, *Cm710*, and *Cl710*. Although *CsCYP87D19* has been implicated in the CuC biosynthetic pathway as part of a gene cluster, its functional characterization is still lacking. Given that *MlCYP87D16*, a close homolog of *CYP87D20*, catalyzes C-16α oxidation of β-amyrin in *Maesa lanceolata* [42], we speculate that *CYP87D20* may perform a similar function in cucurbitacin biosynthesis. *Hel0120370.1* is annotated as *CYP81Q58* and is homologous to *Cs160*, *Cl160*, and *Cm160*; notably, *Cs160* has been functionally characterized to catalyze the C25 hydroxylation of cucurbitacin C (CuC), supporting the hypothesis that *Hel0120370.1* may play a similar role in CuIIa biosynthesis. In contrast, *Hel0120380.1* shows high sequence similarity to *Cs180*, *Cm180*, and *Cl180*, which have been implicated in C2 hydroxylation of cucurbitacin B (CuB) and cucurbitacin E (CuE), respectively, suggesting that *Hel0120380.1* may catalyze the C2 hydroxylation step in the biosynthesis of CuIIa.

In addition, we identified a conserved gene cluster on chromosome 9, designated as Cluster No. 50 (64.15 kb; Table S24), which exhibits 90% structural similarity to a previously reported MATE transporter-associated cluster in cucumber. Homologous clusters were also detected in *B. hispida* and *C. lanatus* with approximately 81% similarity, indicating conserved synteny across species. Specifically, Cluster No. 50 contains two dioxygenases, along with one alcohol dehydrogenase (ADH), two MATE transporters, one CYP450 gene, and one BAHD acyltransferase, constituting a putative functional module involved in triterpenoid biosynthesis, modification, and transport (Fig. 6C). Notably, one MATE transporter shares 73% amino acid identity with a functionally characterized MATE protein from melon that facilitates the efflux of CuB [43], implying a potential role in triterpenoid export or intracellular compartmentalization in *H. ellipsoidea*. Additionally, *Hel0176470.1*, annotated as a member of the CYP87D20 subfamily, shows high sequence similarity to *Cs890*, *Cm890*, and *Cl890*, which have been demonstrated to catalyze C11 carbonylation and C20 hydroxylation of Cuol. This supports its potential involvement in the terminal steps of CuIIa biosynthesis in *H. ellipsoidea*. Although cluster No. 50 does not overlap with the core CuIIa biosynthetic gene cluster, the presence of tailoring enzymes (CYP450 and BAHD) and transporters suggests that it may function in the downstream modification and subcellular trafficking of cucurbitacin-like triterpenoids. Together, the identification of these clusters provides new insights into the genomic basis of triterpenoid biosynthesis in *H. ellipsoidea*. In particular, cluster No. 27 and cluster No. 50 exhibit strong structural conservation and putative functional relevance, offering important implications for understanding the genomic organization and regulatory mechanisms underlying cucurbitacin biosynthesis in this species.

### Genes involved in the biosynthesis of CuIIa in *H. ellipsoidea*

**Figure 7:**
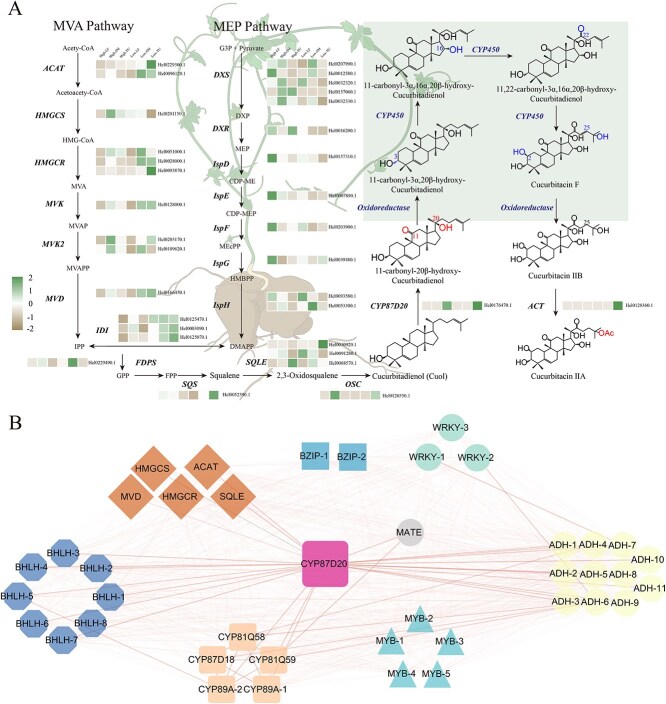
Sampling overview and cucurbitacin biosynthesis in *H. ellipsoidea*. (A) Schematic illustration of tissue sampling sites (leaf, stem, root tuber) from *H. ellipsoidea* individuals under natural growth conditions. (B) Cucurbitacin content in the root tuber of the high-content accession, with an average of 2.5%. (C) CuIIa content in the root tuber of the low-content accession, with an average of 0.5%. (D) Expression profiles of genes involved in CuIIa biosynthesis.

We performed transcriptome sequencing of various tissues from two wild *H. ellipsoidea* individuals under natural growth conditions. Significant differences in cucurbitacin content were observed in the root tubers of these two accessions. After 3 months of continuous monitoring (once per month), the average cucurbitacin content in the high-content root tuber reached 2.5% (Fig. S28), whereas the low-content accession maintained an average of 0.5% (Fig. S29). Based on genome annotation results, we quantified the expression levels of genes involved in the MVA and MEP pathways, as well as other known cucurbitacin biosynthesis-related genes (Fig. 7A). We observed a significant upregulation of MEP pathway genes in the leaves, while MVA pathway genes were predominantly expressed in stems and root tubers. These differences suggest that the biosynthesis of cucurbitacin precursors primarily depends on the MEP pathway localized in chloroplasts of leaf tissue. In contrast, downstream pathway genes exhibited higher expression in stems and root tubers, indicating that cucurbitacin biosynthesis is spatially regulated, with a tissue-specific metabolic flux likely following a ‘leaf-stem-tuber’ directional pattern. Differential expression analysis was conducted on leaves, stems, and root tubers comparing the high and low content accessions. KEGG enrichment analysis of differentially expressed genes (DEGs) revealed that, across all three tissue comparisons (high leaf vs. low leaf, high stem vs. low stem, and high root tuber vs. low root tuber), the significantly enriched pathways consistently included ‘metabolic pathways’ and ‘biosynthesis of secondary metabolites’ (*P* < 0.05; Figs S30–S32). GO enrichment of biological processes (BP) indicated that DEGs in leaves and stems were primarily associated with ‘Catalytic activity’ (Figs S33 and S34). Notably, in stem tissue, several DEGs were also enriched in functions such as ‘beta-amyrin synthase activity’ ‘oxidosqualene cyclase’, and ‘lanosterol synthase activity’, suggesting that variation in triterpenoid biosynthetic enzyme expression in the stem may contribute to cucurbitacin content differences. In root tubers, DEGs were significantly enriched in ‘oxidoreductase activity’, pointing to the possible involvement of redox-related enzymes in late-stage modifications (Fig. S35). These patterns likely reflect distinct metabolic flux control mechanisms in different tissues, contributing to the differential accumulation of cucurbitacins.

Tissue-specific accumulation patterns of cucurbitacins are likely shaped by differential regulation of biosynthetic genes across developmental contexts. Building on previous studies, a putative downstream biosynthetic pathway for CuIIa in *H. ellipsoidea* was proposed. This pathway is initiated from Cuol and proceeds through sequential hydroxylation events followed by a terminal acylation step to produce CuIIa. While candidate genes have been suggested for some enzymatic steps, several key functions, such as C3a-hydroxylation, C16a-hydroxylation, and C22-carbonyl formation, remain uncharacterized. To facilitate the identification of these missing genes, a weighted gene co-expression network analysis (WGCNA) was performed and integrated with pairwise Pearson correlation analysis. Based on transcriptome data from *H. ellipsoidea*, 15 distinct co-expression modules were identified, each grouping genes with similar expression profiles (Fig. S36). We identified 30 genes involved in the upstream biosynthesis of cucurbitacin and classified them into ten co-expression modules based on WGCNA analysis (Table S25). The MEturquoise module contained the largest number of genes (*n* = 8), followed by the MEyellow and black modules (*n* = 5 each), and the MEpurple and MEbrown modules (*n* = 3 each). The MEblue module contained two genes, while the MEred, MEgrey, and MEgreen modules each included one gene. To investigate potential regulatory patterns, we analyzed the correlations between module eigengenes and key trait variables, including CuIIa content, tissue-specific accumulation, and the average expression of genes within clusters No. 27 and No. 50. The MEbrown module was strongly associated with leaf tissue (*P* = 6.4 × 10^−14^), while the MEturquoise module correlated with stem expression (*P* = 2.9 × 10^−7^) (Fig. S37). A significant correlation was also observed between the MEpink module and root tuber samples (*P* = 9.9 × 10^−8^). Notably, the MEyellow module exhibited the strongest positive correlations with CuIIa levels and expression of genes in both gene clusters, whereas the MEpink module showed a similar but weaker trend. Although the *OSC* gene displayed positive associations with the MEtan and MEblue modules, these correlations were not statistically significant, suggesting a potential but unconfirmed regulatory role in upstream triterpenoid biosynthesis. Interestingly, genes involved in cucurbitacin backbone biosynthesis were dispersed across multiple modules, indicating a decentralized regulatory network. In contrast, genes related to downstream modifications were predominantly concentrated in a few specific modules, such as MEyellow, MEblue, and MEtan, implying potential co-regulation during cucurbitacin diversification. Within the MEyellow module, a total of 540 hub genes (GS > 0.7 and MM > 0.7) were identified. Using *CYP87D20* (*Hel0176470.1*) as a bait gene, Pearson correlation analysis was conducted to uncover co-expressed candidates potentially involved in cucurbitacin biosynthesis and regulation(Fig. 7B). This analysis revealed five CYP450 genes, eleven ADH family members, and transcription factors belonging to four distinct families (bHLH, MYB, BZIP, and WRKY) as potential key regulators. Notably, a MATE transporter gene, located adjacent to *Hel0176470.1* within the same biosynthetic gene cluster (cluster No. 50), exhibited a strong expression correlation with the bait gene, suggesting its possible functional involvement in cucurbitacin accumulation.

### The *CYP450* gene family of *H. ellipsoidea*

**Figure 8:**
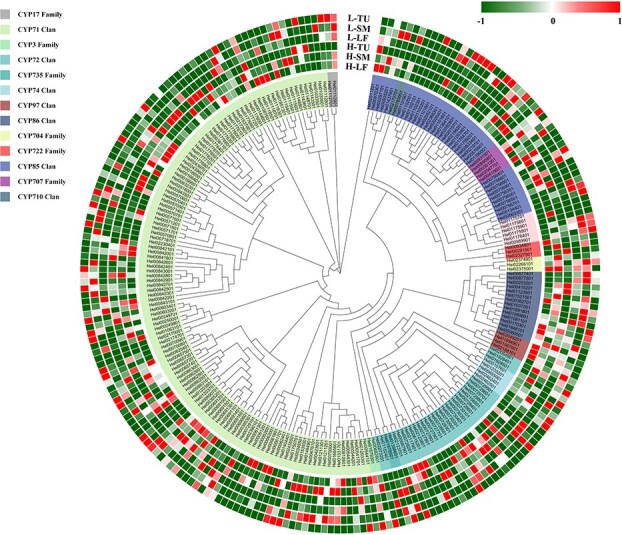
Phylogenetic tree and expression profiles of all CYP450 genes in *H. ellipsoidea*. L-TU, low-tuber lines; L-SM, low-stem lines; L-LF, low-leaf lines; H-TU, high-tuber lines; H-S, high-stem lines; H-LF, high-leaf lines.

In this study, all the CYP450 genes in *H. ellipsoidea* were identified systematically using HMM search in combination with BLAST analysis. Genes from the CYP71, CYP72, CYP85, and CYP86 clans accounted for 82.5% of the total CYP450 genes in *H. ellipsoidea*. Notably, genes annotated as part of the CYP71 clan constituted more than half of the total CYP450 genes, spanning 19 gene families (Table S26). Specifically, we identified 52 CYP450 genes previously reported to be involved in triterpene modification, primarily from the CYP71 clan (20 genes in the CYP71A family and 5 genes in CYP705A), the CYP72 clan (24 genes in the CYP72A family), the CYP85 clan (4 genes in the CYP87D family and 12 genes in the CYP88D family), and the CYP716A (10 genes) and CYP90B families (2 genes). Among the 240 identified CYP450 genes in *H. ellipsoidea*, transcriptome sequencing revealed the expression of 214 genes across three different tissues, as evidenced by the phylogenetic analysis (Fig. 8). The CYP450 genes are distributed across all 14 chromosomes of *H. ellipsoidea*, with a majority of them arranged in tandem arrays on most chromosomes. The highest number of these genes is located on chromosome 10, where a significant proportion are organized in consecutive sequences, forming tandem repeats. Functional annotation and similarity analyses indicate that these genes belong to various gene families, including the CYP71, CYP72, and CYP85 clans. This suggests that, despite largely preserving its original karyotype during extensive evolutionary processes, chromosome 10 of *H. ellipsoidea* has experienced substantial changes in both the copy number and functional diversification of its hydroxylase genes.

Additionally, all the CYP450 genes from the genomes of *C. sativus*, *C. lanatus*, and *C. melo* were analyzed together using published data [12, 19] These species are capable of synthesizing Cuol, which acts as an intermediate compound for the production of CuIIa. The differences in the number of CYP450 genes among *H. ellipsoidea* (240 genes), cucumber (214 genes), watermelon (208 genes), and melon (228 genes) further highlight the diversity within this gene family across these species. Orthofinder [44] analysis was employed to cluster CYP450 genes into orthologous groups, revealing distinct gene orthogroups that may contribute to functional diversification. Among the 890 CYP450 genes analyzed across four species, 231 orthogroups were identified, with 157 orthogroups containing at least two CYP450 genes (Table S25). Furthermore, a subset of CYP450 genes exists as single-copy genes that are species-specific, with no homologs detected in the other three species. Notably, 51 CYP450 genes were exclusively present in the genome of *H. ellipsoidea*, and these genes predominantly belong to the CYP85 clan, CYP86, the CYP71 clan, and the CYP72 family. Among the 10 sequences annotated as members of the CYP85 clan, transcripts from seven genes were detected to be expressed in root tubers. These *H. ellipsoidea*-specific genes may potentially perform novel functions and catalyze the synthesis of triterpenoid products that differ from those in other species.

## Discussion

In contrast to most genera in the Cucurbitaceae family, *Hemsleya* forms a distinct evolutionary branch characterized by adaptations associated with medicinal properties. The root tuber of *Hemslyea* genus is rich in nutrients and demonstrates significant potential for the biosynthesis of high concentrations of cucurbitacins. It is regarded as a valuable source for pharmaceutical extraction due to its ability to specifically synthesize cucurbitacin IIa. To date, at least 11 genera of the Cucurbitaceae family have been sequenced, including *Cucumis* [33, 45, 46], *Benincasa* [26, 38], *Cucurbita* [24, 47, 48], *Lagenaria* [30], *Momordica* [27], *Citrullus* [32, 49], *Luffa* [50, 51], *Siraitia*, *Sechium,* and *Trichosanthes*, and are available through the Cucurbitaceae genome database [52]. Over the course of long-term evolution, most species in the Cucurbitaceae have been domesticated into vegetables or fruits; however, a few, such as *H. ellipsoidea*, *Trichosanthes kirilowii* [53], and *Citrullus colocynthis* [54], have retained a primary focus on their high medicinal value. However, genomic resources for these medicinally important taxa remain scarce, constraining comprehensive insights into the evolutionary history of the family and impeding detailed elucidation of terpenoid biosynthetic pathways in Cucurbitaceae plants. In recent years, more traditional Chinese medicinal plants have gained recognition following the completion of their genome assemblies. The genome of *Tripterygium wilfordii* has facilitated the identification and characterization of CYP728 family genes involved in the biosynthetic pathway of tripterygium glycosides [28]. The genome of *ginseng* elucidates a significant relationship between tandem duplications and the biosynthesis of ginsenosides [55]. The genome of *Centella asiatica* has enabled the identification of key gene families and unique gene clusters involved in triterpenoid saponin biosynthesis, providing a foundation for metabolic engineering and molecular breeding [56]. To further investigate the biosynthetic pathways of terpenoid secondary metabolites in Cucurbitaceae plants and to uncover more potential genes, we have successfully completed the high-quality genome assembly of *H. ellipsoidea* for the first time. These results not only enhance our understanding of the evolutionary history of Cucurbitaceae plants but also provide new insights into the biosynthesis of cucurbitacins.

In this study, we assembled the high-quality chromosome-level genome *H. ellipsoidea*, consisting of 14 chromosomes and totaling 535.68 Mb, which is larger than that of most reported Cucurbitaceae species. This study presents the first reported genome of a medicinal plant within the Cucurbitaceae family. Although the number of protein-coding genes is comparable to those of other Cucurbitaceae species, the genome displays a notably higher level of heterozygosity (1.62%), indicating that *H. ellipsoidea* possesses greater genetic diversity relative to domesticated species within the family. Such diversity provides a valuable genetic reservoir for elucidating adaptive traits and for future breeding efforts. In order to better understand the evolutionary history of *H. ellipsoidea*, we conducted a comparative analysis of its phylogenetic relationships with other species within the Cucurbitaceae family. Our findings indicate that *Hemsleya* genus diverged from other Cucurbitaceae species as early as approximately 84.7 million years ago. Evolutionary analyses indicate that the *Hemsleya* genus diverged from other Cucurbitaceae lineages during an early stage of the family's diversification and has not undergone any subsequent WGD events. This deep divergence underscores its distinct evolutionary trajectory within Cucurbitaceae and provides a unique framework for investigating lineage-specific adaptations and specialized metabolite biosynthesis.

The *B. hispida* genome serves as the most ancestral-like reference for Cucurbitaceae. Our comparative analysis places *H. ellipsoidea* as phylogenetically close to this ancestral state, yet distinct due to specific structural innovations. The quantitative summary of chromosomal rearrangements reveals a mosaic pattern of evolution: extreme conservation coexists with extensive reshuffling. The complete preservation of Chr 9 (derived from Ancestral Chr 6) suggests strong purifying selection, implying that the gene order on this chromosome likely governs conserved biological functions essential for Cucurbitaceae survival. In contrast, the extensive fragmentation observed in Chr 2 (16 blocks) and Chr 1 (fused sources) indicates evolutionary hotspots where genomic plasticity is maximized. These extensive rearrangements likely drove the ecological divergence of *H. ellipsoidea*. Specifically, the ‘shattering’ of ancestral chromosomes allows for the breakage of linkage drag, facilitating the recombination of alleles that were previously genetically linked. This reshuffling creates new regulatory landscapes, potentially bringing biosynthetic gene clusters—such as those controlling CuIIa synthesis—under new promoter control or grouping them for co-expression. We hypothesize that this structural plasticity provided the genetic raw material for *H. ellipsoidea* to adapt to high-altitude stress and biotic pressures. The contrast between the static Chr 9 and the dynamic Chr 2 highlights a strategy where the genome maintains core functions while simultaneously permitting localized hyper-evolution to drive speciation and medicinal trait diversification.

Terpenoid biosynthesis, including triterpenoids, is typically organized into three distinct stages. The first stage involves the formation of the universal C_5_ isoprenoid precursors, IPP and DMAPP, through either the MVA pathway or the MEP pathway. In the second stage, these precursors undergo sequential condensation and cyclization, often mediated by OSCs, to construct the core triterpenoid skeleton. The third stage comprises a series of structural modifications—such as oxidation (e.g., hydroxylation), glycosylation, acetylation, and reduction that diversify the backbone into various bioactive derivatives with distinct biological activities [57]. CuIIa content is a key indicator for assessing the medicinal quality of *H. ellipsoidea*. Therefore, elucidating its biosynthetic pathway and identifying key enzyme-coding genes are essential for promoting crop breeding and genetic improvement. Based on previous research, we reconstructed the CuIIa biosynthetic pathway and identified candidate genes involved in its synthesis. In the MVA pathway, most associated genes were found to be multi-copy, whereas in the MEP pathway, only two genes were duplicated and the rest existed as single copies. A similar pattern of copy number has been observed in other Cucurbitaceae species, such as watermelon [58], where most MEP-pathway genes are also present as single copies. Although exceptions like the DXS gene, which may have a few paralogs, have been reported, the trend remains largely consistent. In addition, phylogenomic analyses across land plants have shown that six out of seven canonical MEP-pathway genes are typically maintained as single-copy genes, even after multiple rounds of WGD [59]. This pattern suggests strong evolutionary constraints and high functional conservation. Our findings in *H. ellipsoidea* are consistent with these observations and may reflect the essential roles of MEP-pathway genes in primary metabolism.

Transcriptomic analysis revealed clear tissue-specific expression patterns of triterpenoid biosynthetic genes in *H. ellipsoidea*, with MVA pathway genes predominantly active in stems and root tubers, whereas MEP pathway genes were more highly expressed in leaves. Moreover, genes involved in the downstream modification processes of CuIIa biosynthesis—particularly cytochrome P450 and BAHD acyltransferases exhibited high expression almost exclusively in root tubers. Based on the observed spatial expression patterns of key biosynthetic genes, we propose a compartmentalized model for CuIIa biosynthesis in *H. ellipsoidea*. The precursors are likely synthesized in the leaves, followed by the formation of Cuol in stem tissues. Subsequently, these intermediates may be transported through the vascular system to the root tubers, where the final structural modifications, such as cytochrome P450 mediated oxidation and BAHD acyltransferase-catalyzed acylation. This implies that the root tubers serve as the principal sites for both the terminal biosynthetic steps and the storage of CuIIa. This spatial organization contrasts with findings in cucumber, where CuC was reported to be synthesized in mesophyll cells and transported to specific storage sites via the *CsMATE1* transporter [60]. Moreover, recent advances in metabolite-based genome-wide association studies (mGWAS) have demonstrated the power of integrating metabolic and genomic datasets to identify key genes underlying specialized metabolite biosynthesis, as exemplified in tea plant (*Camellia sinensis*) [61]. Such integrative frameworks could be further applied to *Hemsleya* species to dissect the genetic basis of cucurbitacin diversity and to accelerate functional gene discovery and metabolic improvement.

Previous studies have identified gene clusters in plants that regulate the biosynthesis of secondary metabolites, such as those found in rice [62] and *Salvia miltiorrhiza* [63]. In this study, we also discovered candidate gene clusters in the tepernoid biosynthetic pathway, among which two clusters are closely associated with the biosynthesis of CuIIa. Cluster No. 27 shared 88% structural similarity with a previously characterized CuC biosynthetic gene cluster in *C. sativus* [33], and exhibited conserved synteny with homologous clusters in melo and watermelon [19]. Cluster No. 50 contains both a candidate cytochrome P450 hydroxylase gene and a MATE-type transporter gene, suggesting its potential involvement in the terminal modification and intracellular transport of CuIIa. This cluster is also conserved in cucumber, melo, and watermelon. Notably, a recent study demonstrated that in melo, root epidermal cells utilize a MATE transporter to secrete cucurbitacins into the rhizosphere, modulating root-associated microbial communities, and enhancing resistance to fusarium wilt [43]. These findings further demonstrate that the gene cluster-based strategy for secondary metabolite biosynthesis is a conserved feature among Cucurbitaceae species. The conserved presence of this clusters implies coordinated regulation of both synthesis and compartmentalization of bioactive triterpenoids. Furthermore, transcriptomic analyses revealed that genes within these clusters exhibit highly similar expression patterns, reinforcing their functional coherence. Taken together, the discovery and characterization of these conserved clusters not only deepen our understanding of cucurbitacin biosynthesis but also provide valuable targets for future functional validation and metabolic engineering aimed at enhancing the production of bioactive compounds in medicinal cucurbits.

The CYP450 gene family constitutes approximately 1% of plant-encoded proteins and represents the largest enzyme family involved in plant specialized metabolism [64]. The enzymes play indispensable roles in the triterpenoid biosynthesis, typically catalyzing modification steps following the completion of triterpene backbone synthesis [65]. Notably, CYP450 genes exhibit remarkable substrate specificity across organisms. At present, the botanical kingdom demonstrates a collective presence of 127 distinct families attributed to the CYP450 group [66], well in terrestrial plants, a total of 11 cytochrome P450 (CYP) clans have been identified [67]. Among these, the CYP51, CYP71, CYP72, and CYP85 clans are believed to be involved in the structural modification of triterpenes. The cytochrome P450 gene family in *H. ellipsoidea* is markedly expanded toward these clans, reflecting a strategic investment in late stage modifications that determine CuIIa's bioactivity and compartmentalization, while upstream precursor pathways remain evolutionarily constrained. The prevalence of tandemly duplicated CYPs suggests a mechanism by which catalytic diversity is generated through local gene duplication and neofunctionalization, a pattern consistent with CYP evolution in diverse plant lineages [68]. *H. ellipsoidea* also possesses lineage-specific CYPs drawn predominantly from triterpenoid-modifying clans, distinct from its cucurbit relatives, suggesting that species-specific metabolite profiles arise through redeployment of oxidative capacities. Indeed, similar biosynthetic frameworks in cucurbits are differently populated by CYP inventories, yielding unique chemotypes [19]. A compelling aspect of this tailored toolkit is its apparent genomic coupling. Cluster No. 50, which co-locates candidate CYPs and a MATE transporter, aligns with emerging evidence in melon where cucurbitacin secretion into the rhizosphere via MATE proteins modulates root-associated microbiomes and contributes to disease resistance. This co-localization supports a model where biosynthesis, modification, and export are integrated at the gene-cluster level. These findings suggest that *H. ellipsoidea* has evolved a lineage-specific oxidative module to efficiently complete CuIIa biosynthesis, particularly in underground tissues. Family-wide comparisons highlight that while the biosynthetic framework is conserved, CYP expansions vary, underpinning chemotypic divergence. Thus, metabolic engineering or breeding should prioritize tailoring enzymes at pathway termini, which represent critical nodes for modulating CuIIa accumulation and specificity.

## Materials and methods

### Plant materials

The Wild *H. ellipsoidea* plants were collected in Lingcang, Yunnan, China. The species was identified through morphological observations and comparing with the records in the *Flora of China.* Young and tender leaves were sampled for genomic DNA extraction. Each accession was carefully labeled and recorded for subsequent analyses. In August, additional tissues including leaves, stems, and root tubers were collected and flash-frozen in liquid nitrogen, which were subsequently used for RNA extraction and quantification of CuIIa content.

### Whole genome sequencing and RNA sequencing

For whole-genome sequencing, genomic DNA was obtained from *H. ellipsoidea* leaf buds using a modified CTAB extraction procedure [69]. Illumina paired-end sequencing libraries were prepared with an average insert length of 150 bp. After filtering the low-quality reads, a total of 56.07Gb paired-end sequences was generated on sequencing platform Illumina NovaSeq 6000 platform. For long-read sequencing, libraries were constructed using the SQK-LSK109 kit (Oxford Nanopore Technologies) and sequenced on the PromethION P48 platform (Oxford, UK). The raw data were then transferred into fast5 format through Oxford Nanopore GUPPY (version 4.0.2). In addition, Hi-C libraries were constructed and sequenced using the Illumina platform, and the data obtained were applied to Hi-C analysis.

RNA sequencing was performed to assist gene annotation and expression analysis. Total RNA was extracted from leaves, stems, and root tubers of *H. ellipsoidea* (three biological replicates per tissue) using TRIzol reagent (Invitrogen, USA). RNA quality was assessed with a NanoDrop One spectrophotometer and a Qubit 3.0 Fluorometer. mRNA was isolated from total RNA using Oligo (dT) magnetic beads and subsequently fragmented in fragmentation buffer to construct RNA pools. cDNA libraries were generated with the NEBNext® Ultra™ RNA Library Prep Kit for Illumina® (New England Biolabs, USA). Amplified products were purified using the AMPure XP system, and library quality was evaluated on an Agilent 2100 Bioanalyzer. The libraries were sequenced on the Illumina NovaSeq 6000 platform, producing 150-bp paired-end reads.

### K-mer analysis and genome assembly

Genome characteristics, including genome size and heterozygosity, were assessed using GCE (v1.0.2) [70]. and genomescope (version 1.0.0) [71] based on a 19-K-mer distribution. The ONT nanopore sequences were firstly assembled by nextDenovo [72].

To improve assembly accuracy, the initial assembly was polished twice with Racon (v1.4.11) and subsequently refined using Pilon (v1.22) [73] with Illumina NovaSeq data. For chromosomal scaffolding, Hi-C raw reads were processed with HiCUP (v0.7.2) [74], and the high-quality reads were then mapped onto the preassembled contigs. Following the removal of misaligned reads, the processed Hi-C data were analyzed with ALLHiC (v0.9.12) [ 75], which was used to cluster, order, and orient the contigs for the construction of draft pseudochromosomes. For quality assessment, the pseudochromosome assemblies were segmented into 150-kb bins of equal length, and interaction frequencies between bins, derived from validly mapped Hi-C read pairs, were visualized in a heat map. The overall completeness and accuracy of the final genome assembly were subsequently examined with BUSCO (v4.1.2) [76].

### Gene structure and function annotation

In the *H. ellipsoidea* genome, de novo transposable elements were first predicted with RepeatModeler (v1.0.11) and used to construct a custom repeat library. Repeats were then masked and classified into families using RepeatMasker (v4.0.9). Gene prediction was carried out on the masked assembly by combining transcript mapping, ab initio prediction, and homology-based evidence. For homology-based prediction, protein sequences from five species (*C. melo*, *M. charantia*, *C. maxima*, *C. pepo*, *B. hisp*ida) were used to align the genome sequences through Exonerate (version:v2.4.0). Ab initio predictions were generated with Augustus (v3.3.2), Genscan (v1.0), and GlimmerHMM (v3.0.4). To provide transcript evidence, RNA-seq data from three tissues were aligned to the *H. ellipsoidea* genome using STAR (v2.7.9a) [77] and assembled with StringTie (v2.1.4) [78]. MAKER (v2.31.8) was employed to integrate ab initio, transcript-based, and homology-based evidence. The completeness and quality of the final gene models were subsequently assessed with BUSCO (v4.1.2).

The anticipated gene models were subjected to functional annotation utilizing Blast(version:2.6.0+) software, aligned against Uniprot (http://www.uniprot.org/), Pfam (pfam.xfam.org/), Gene Ontology (GO) [79], Kyoto Encyclopedia of Genes and Genomes (KEGG) (http://www.genome.jp/kegg/), InterPro [80] (https://www.ebi.ac.uk/interpro/), Nr (non-redundant database), databases. Annotation of non-coding RNAs was performed using multiple approaches: tRNA genes were detected with tRNAscan-SE (v1.3.1; http://lowelab.ucsc.edu/tRNAscan-SE/) [81], rRNAs were predicted with RNAmmer (v1.2), and additional ncRNA families were identified through INFERNAL (v1.1.2) [82].

### Gene families and phylogenetic analysis

To examine gene family evolution, Orthofinder (v2.5.4) [44, 83] was applied to the *H. ellipsoidea* genome together with those of 10 additional species (*C. sativus*, *C. melo*, *B. hispida*, *C. moschata*, *C. pepo*, *C. maxima*, *M. charantia*, *V. vinifera*, *O. sativa*, and *A mborella trichopoda*). The sequence pairs’ similarities were assessed using BLASTP with a predefined threshold e value of 1e−5. Following this, gene families were clustered using OrthoMCL (version 2.0.9) with its default settings.

The protein sequences of each single-copy gene family were aligned using the software Muscle (version 5.1). Sequence alignments were processed with trimAl (v1.4.rev22; −gt 0.2) to remove poorly aligned regions, and the resulting high-quality alignments were concatenated into a supermatrix for phylogenetic inference. A Maximum Likelihood tree was constructed using RAxML (v8.2.10) [84] under the PROTGAMMAWAG substitution model. Divergence time estimation was conducted with MCMCtree in PAML (v4.9) [85], using parameters *nsample = 1 000 000*, *burnin = 200 000*, *seqtype = 0*, and *model = 4*, with calibration points obtained from TIMETREE (http://www.timetree.org/). Based on the orthologous gene families combined with divergence times, Cafe software (version 4.2.1) [86] was applied to infer patterns of gene family expansion and contraction across species.

### Genome evolution of *H. ellipsoidea*

To infer ancestral karyotypes of cucurbit plants, we adopted the strategy reported in the *B. hispida* genome sequencing study [26]. Homologous and collinear genes were identified, and intra- genomic and inter-genomic dot plots were generated using WGDI with the -d command. Subsequent fusion and fission events underlying the evolution of *H. ellipsoidea* chromosomes from the ancestral karyotype were inferred following the WGDI pipeline.

### Collinearity and whole-genome duplication analysis

The analysis of Ks distribution was employed to deduce instances of WGD in *H. ellipsoidea*. This involved examining paralogous gene pairs within the species. Additionally, the comparison of genetic divergence between species was conducted by analyzing orthologous genes to further understand the occurrence of WGD in *H. ellipsoidea*. BLASTP was used to align protein sequences both within each genome and across different genomes. Gene pairs with an e-value below 1e−5 were regarded as potential homologs, and syntenic blocks were subsequently defined using WGDI [87]. Synteny within the genome was analyzed to detect potential WGD events. Ks values (synonymous substitutions per synonymous site) were calculated with the YN00 method in WGDI (−ks option). Figures were produced following the WGDI pipeline using the -kp, −pf, and -kf commands.

### Investigation of the Genge family involved in the biosynthesis pathway of Cucurbitacin IIa

Identification of candidate homologs was carried out through HMM-based searches [88] and BLASTP analysis. Reference protein sequences corresponding to 17 key genes (*A. thaliana* ACAT, CMK, DXR, DXS, FPS, GGPPS, GPS, HDR, HDS, HMGR, HMGS, IDI, MCT, MCS, MVK, MVD, and PMK) were downloaded from the NCBI database and employed as queries. BLASTP searches were performed against the *H. ellipsoidea* genome using an E-value cutoff of 1e−5, and only hits with ≥50% coverage of the seed proteins and > 50% sequence identity were retained. Candidate sequences were aligned with MAFFT [89], and phylogenetic reconstruction was performed using PhyML [90].

### CYP450 gene family identification and phylogenetic tree building

The HMM profile (PF00067) was downloaded from Pfam (pfam.xfam.org/). The HMM search was used to obtain the homologue CYP450 genes in the protein database of *H. ellipsoidea.* Thus, All CYP450 genes were systematically enumerated and categorized into distinct families and subfamilies. Reference gene clusters from *C. sativas, C. lantuas*, and *C. melo* were utilized as a basis for conducting a protein database search within *H. ellipsoidea* using the Blast software. All the CYP450 genes were aligned using MAFFT software, then IQ-TREE2 [91] was used to build a phylogenetic ML tree with the best-fit models VT + F + R6. The CYP450 genes and gene clusters associated with triterpene synthesis were mapped onto chromosomes and visualized using TBtools [92].

### Measurement of Cucurbitacin IIa content (HPLC)

Methanol extracts of *H. ellipsoidea* were clarified by 0.22-μm filtration and analyzed by HPLC. Separation was achieved on a C18 column (250 × 4.6 mm, 5 μm) operated at 30°C. The mobile phase consisted of water (A) and acetonitrile (B) under a gradient of 75% to 50% A (0–20 minutes), 50% to 20% A (20–30 minutes), 20% to 75% A (30–35 minutes), and 75% A (35–40 min) for re-equilibration. The chromatographic conditions included a flow rate of 1.0 mL/min, injection volume of 10 μL, and UV detection at 210 nm.

### Compliance with ethics requirement

This article does not contain any studies on human or animal subjects.

## Supplementary Material

Web_Material_uhaf363

## Data Availability

The *H. ellipsoidea* genome and raw transcriptome sequencing data generated in this study have been deposited in the China National GeneBank Sequence Archive (CNSA) under the project accession number CNP0008602. All data will be publicly accessible upon publication.
